# Diagnostic and Prognostic Value of Salivary Biochemical Markers in Oral Squamous Cell Carcinoma

**DOI:** 10.3390/diagnostics10100818

**Published:** 2020-10-14

**Authors:** Lyudmila V. Bel’skaya, Elena A. Sarf, Denis V. Solomatin, Victor K. Kosenok

**Affiliations:** 1 Biochemistry Research Laboratory, Omsk State Pedagogical University, 14, Tukhachevsky str, 644043 Omsk, Russia; nemcha@mail.ru; 2 Department of Mathematics and Mathematics Teaching Methods, Omsk State Pedagogical University, 14, Tukhachevsky str, 644043 Omsk, Russia; denis_2001j@bk.ru; 3 Department of Oncology, Omsk State Medical University, 12, Lenina str, 644099 Omsk, Russia; victorkosenok@gmail.com

**Keywords:** saliva, oral squamous cell carcinoma, biochemical markers

## Abstract

The purpose of the work is a comprehensive assessment of biochemical saliva markers for the diagnosis and prognosis of oral cancer. The group of patients included 68 patients with oral squamous cell carcinoma, 50 with non-cancerous diseases of the oral cavity, and 114 healthy volunteers. Before the start of treatment, 23 biochemical parameters of saliva were determined. Participants were monitored for six years to assess survival rates. The statistical analysis was performed by means of Statistica 10.0 and R package. A complex of metabolic changes occurring in saliva in oral cancer is described. It was shown that none of the studied parameters could be used to diagnose oral cancer in an independent variant; the use of combinations of parameters is more informative. The high prognostic value of the content of malondialdehyde (MDA) and the Na/K-ratio in saliva before treatment was established. Thus, the content of MDA ˂ 7.34 nmol/mL and the Na/K-ratio > 1.09 c.u. is a prognostically unfavorable factor (HR = 7.88, 95% CI 1.10–54.62, *p* = 0.01876), which may be useful for optimizing the treatment of patients with oral cancer. It has been shown that saliva has great potential for the development of diagnostic and prognostic tests for oral cancer.

## 1. Introduction

Cancer of the oral cavity and oropharynx takes the 8th place in the structure of the general incidence of malignant neoplasms and the 1st among the malignant tumors of the head and neck (excluding non-melanoma skin lesions). Oral squamous cell carcinoma (OSCC) is diagnosed in 90% of all cases of oral cancer [1]. Almost 300,000 new cases of the disease are observed every year in the world [2], of which 145,000 result in the death of the patient [3]. The overall five-year survival rate in such patients does not exceed 60%; the risk of relapse is 30% [4]. The peak incidence occurs at the age of about 60 years, but in the younger group in recent years, there has been an increase in the detection of oropharyngeal cancer associated with human papillomavirus (HPV) [5]. In Russia, the number of OSCC patients with stage III and IV is 61.7% and 81.1%, respectively, of the number of all tumors of this localization [6]. Therefore, the search for new methods for the early diagnosis of oral cancer remains an urgent task to date [7].

In connection with the visual localization of tumors of the oropharyngeal zone, the primary diagnosis is most often established based on examination and biopsy data [8]. In the current international practical recommendations, the standard diagnostic method is a computer tomography (CT) and/or magnetic resonance imaging with intravenous contrast [9]. Various studies are devoted to the use of “omics” technologies, including genomic, transcriptomic, proteomic and metabolic profiling for the detection of biomarkers of oral cancer in tissues, blood, cell lines, urine, etc. [10,11,12]. However, the results obtained are currently not included in clinical recommendations and are not used in clinical practice.

It is logical to assume that the use of saliva is most promising for the detection of oral cancer. Obtaining saliva samples is non-invasive; there is a lower risk of infection, while direct contact of saliva with oral pathologies can contribute to the earlier detection of relevant diseases [13,14]. More than 100 biomarkers have shown differential levels in saliva of OSCC patients [1,15,16]. They include a large number of proteins that encompass cell surface molecules (CD44sol, CA-125) [17,18]; cytoskeletal fragments (CYFRA 21-1) [19]; intracellular proteins (ZNF-510, Mac-2 binding protein) [20,21]; proteases (MMP, cathepsin V, kallikrein 5 and ADAM9) [22,23]; inflammation-related proteins (CRP, defensin-1) [24,25]; mRNA signatures (DUSP1, OAZ1, SAT and H3F3A) [26], and some non-coding RNAs (miRNA, in particular miR-9, miR-134 and miR-191, and circular RNA) [27,28,29]. IL-1b, IL-6, IL-8, MIP-1b, eotaxin and IFN-g and TNF-a showed significant differences between OSCC patients and controls [30]. Salivary-mutated and salivary-methylated DNA, HPV DNA, telomerase levels, specific microbiota of the oral cavity [31], biomarkers of metabolic and oxidative stress [32] and inorganic ion concentration [33] demonstrated the potential of biomarkers. In a number of studies, the content of volatile organic compounds was also determined [34].

The prognostic significance of various factors has been studied in a sufficient number of studies, but at the moment, unfortunately, no single indicator or combination thereof has been found that unequivocally allows us to assess the prognosis of the course of the disease and the risk of its relapse [24,35,36].

The aim of the study was a comprehensive assessment of the biochemical markers of saliva in order to diagnose and predict the course of oral cancer.

## 2. Materials and Methods

### 2.1. Study Design

The study involved volunteers who were divided into three groups: the main, the comparison and the control groups. The main group included patients with a histologically confirmed diagnosis of oral cancer (n = 68, including larynx and nasopharynx—32, oral mucosa—21, tongue—15; in all cases, the histological type of tumor is squamous cell carcinoma, stages I–IV according to UICC TNM staging system, 7th edition). The comparison group included patients with benign tumors of the oral cavity (n = 50, including polyps—6, papillomas—8, cysts—6, adenomas—30). The control group included patients without pathologies of the oral cavity and significant systemic pathologies, including diabetes mellitus, oncological diseases, etc. (n = 114). The inclusion criteria were considered: the age of patients 30–75 years, the absence of any treatment at the time of the study, including surgery, chemotherapy or radiation, the absence of signs of active infection (including purulent processes), the absence of inflammatory diseases of the oral cavity and periodontium. Exclusion criteria: lack of histological verification of the diagnosis. The patients of the main group and the comparison group did not have concomitant diseases that could affect the biochemical composition of saliva, including diabetes mellitus, inflammatory diseases, autoimmune diseases, etc.

The study was approved at a meeting of the ethics committee of the Clinical Oncology Center in Omsk dated 21 July 2016, protocol No. 15.

### 2.2. Collection and Preparation of Saliva Samples

All participants had saliva sampling before starting treatment. Saliva samples (5 mL) were collected on an empty stomach after rinsing the oral cavity with water in the range of 8–10 h in the morning by spitting in sterile polypropylene tubes, the salivation rate (mL/min) was calculated. Saliva samples were centrifuged (10,000× *g* for 10 min) (CLb-16, Moscow, Russia). Biochemical analysis of saliva was performed immediately without storage and freezing [37].

### 2.3. Biochemical Analysis of Saliva Samples

In all saliva samples, 23 biochemical parameters were determined [38]. The concentration of potassium and sodium ions (mmol/L) was determined using the KAPEL-105M capillary electrophoresis system (Lumex, St. Petersburg, Russia); Ca/P and Na/K-ratio were calculated [39]. The total calcium content (mmol/L) was determined photometrically on a StatFax 3300 semi-automatic biochemical analyzer (Awareness Technology, Palm City, FL, USA) by reaction with Arsenazo III, phosphorus (mmol/L) by the reaction of molybdenum acid ammonium, chlorides (mmol/L) by reaction with mercury thiocyanate. Urea concentration (mmol/L) was determined photometrically by the Bertlot urease-salicylate method, total protein (g/L) by reaction with pyrogallol red, albumin (g/L) by reaction with green bromocresol, uric acid (UA, nmol/mL) by uricase method, sialic acids (SA, mmol/L) according to the Hess method. Alkaline phosphatase activity (ALP, U/L) was determined by the Bessey–Lowry–Brock endpoint method, lactate dehydrogenase (LDH, U/L) by the kinetic ultraviolet method according to the NADH (Nicotinamide Adenine Dinucleotide) oxidation rate, gamma glutamyl transferase (GGT, U/L) kinetic method using L-gamma-glutamyl-3-carboxy-4-nitroanilide as a Zeitz–Persin substrate, α-amylase (U/L) by kinetic method for the hydrolysis of CNP-oligosaccharide with the formation of 2-chloro-4-nitrophenol. The activity of superoxide dismutase (SOD, c.u.) was determined by the accumulation of the product of auto-oxidation of adrenaline by the superoxide anion radical in an alkaline medium, catalase (CAT, mcat/L) by the rate of decrease of hydrogen peroxide in the incubation medium. The content of substrates for lipid peroxidation processes (diene conjugates—DC, triene conjugates—TC, Schiff bases—SB, c.u.) was determined spectrophotometrically by the Volchegorsky method. The content of malondialdehyde (MDA, nmol/mL) was determined by reaction with thiobarbituric acid. The level of middle molecular toxins was determined by ultraviolet spectrophotometry at wavelengths of 254 and 280 nm, the ratio MM 280/254 nm was calculated (MM, c.u.).

### 2.4. Statistical Data Processing

Statistical analysis was performed using the programs Statistica 13.3 EN (StatSoft, Tulsa, OK, USA); R version 3.6.3; RStudio Version 1.2.5033; FactoMineR version 2.3. (RStudio, version 3.2.3, Boston, MA, USA). The sample was described nonparametrically using the Mann–Whitney U-test and the Kruskal–Wallis H-test by calculating the median (Me) and interquartile range in the form of the 25th and 75th percentiles (LQ; UQ). Differences were considered statistically significant at *p* < 0.05.

Checking whether a pair of sets of values of the observed indicator belongs to one class was performed using the Wilcoxon pair test at a significance level of 0.05. To search for blocks of pairwise equivalent data, denoted by the same letters on the graphs, the Bron–Kerbosch algorithm was used. A principal component analysis (PCA) was performed using the PCA program in R [40]. The significance of the correlation is determined by the correlation coefficient (r): strong—r = ±0.700 to ±1.00, medium—r = ±0.300 to ±0.699, weak—r = 0.00 to ±0.299. The rate of change of individual biochemical parameters was quantified using the ratio of natural logarithms (LnRR), with LnRR (indicator) = ln (OSCC or Comparison/Control value). Medians and interquartile range presented in figures were calculated using non-transformed data.

Kaplan-Meier method was applied to calculate survival curves and comparison was made using the Log-rank test for univariate analysis (Statistica 10.0, StatSoft). Prognostic factors were analyzed by multivariate analysis using Cox’s proportional hazard model. Overall survival (OS) was computed from the date of diagnosis to the date of death (Complete) or the date of last follow-up (Censored). Survival data were obtained until December 2019.

## 3. Results

### 3.1. Biochemical Saliva Markers in the Diagnosis of OSCC

At the first stage, the biochemical composition of the saliva of healthy volunteers and patients with malignant and non-malignant neoplasms in the oral cavity was compared (Table 1). It was shown that against the background of pathological processes in the oral cavity, an increase in the concentration of electrolyte components of saliva is observed, while the protein content decreases. A statistically significant increase in the activity of metabolic enzymes was established for both the main group and the comparison group, however, the activity of catalase in both cases decreases compared to the control group. An increase in the content of lipid peroxidation products (diene, triene conjugates and Schiff bases) was also noted, while the concentration of MDA remained practically unchanged. The ratio MM is also growing, which indicates an increase in the level of endogenous toxins.

**Figure 1:**
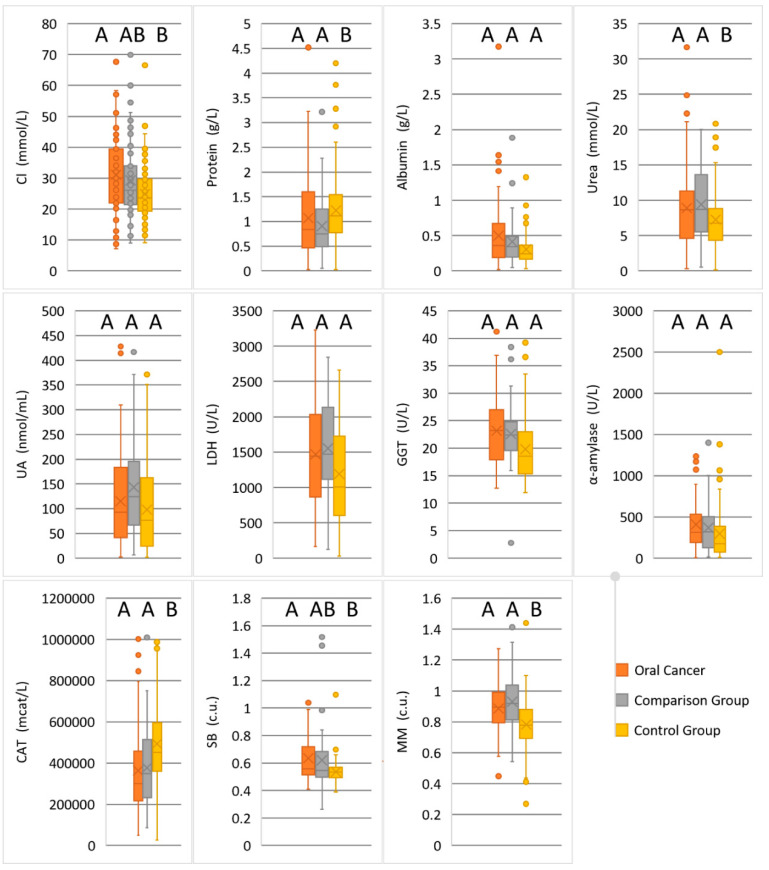
Comparison of statistical characteristics in the studied groups. The cross is mean; the rectangle corresponds to the first and third quartiles. The letters indicate the differences between the groups. Means without a common letter statistically differ (*p* < 0.05).

For indicators that demonstrate differences between the studied groups (Kruskal–Wallis criterion, Table 1), diagrams are additionally constructed (Figure 1). It was shown that the maximum differences are observed between the control group and patients with pathologies of the oral cavity (both benign and malignant). Differences between the three groups are confirmed for chlorides and Schiff bases (Groups A, AB and B, Figure 1).

**Figure 2:**
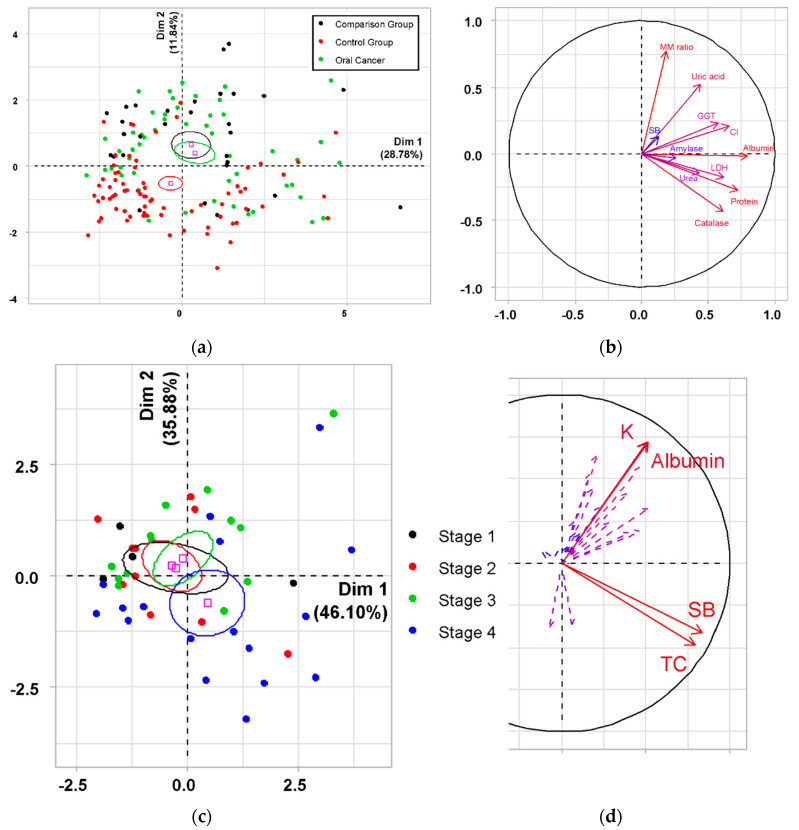
The factor plane (**a**) and the correlation circle (**b**) for groups of oral cancer group, comparison group and control group. The factor plane (**c**) and the corresponding correlation circle (**d**) by stages of oral squamous cell carcinoma (OSCC). On the correlation circle, the variables are sorted by importance level in color from blue (0) to red (1).

The data analyzed by the PCA method contained 232 records with 11 quantitative and 1 qualitative variables in each, which is quite representative. The inertia of the first measurements shows whether there are strong relationships between the variables and indicates the number of measurements that need to be studied. The first two measurements cover 40.62% of the total inertia of the data set (28.78% of the first axis and 11.84% of the second axis); this means that 40.62% of the total variability is explained by two PCs. This percentage is relatively high and, therefore, the two-dimensional plane well represents the variability of the data (Figure 2a). As a result of PCA, we obtained the distribution of parameters along the axes of the factor plane (Figure 2b, *p* < 0.05). For PC1 (horizontal axis), high correlation coefficients were obtained for albumin (*r* = 0.80) and protein (*r* = 0.72), medium strength correlations for chlorides (*r* = 0.65), LDH (*r* = 0.62), catalase (*r* = 0.61), GGT (*r* = 0.58), uric acid (*r* = 0.44) and urea (*r* = 0.42). For PC2 (vertical axis), a high correlation coefficient was noted only for the ratio MM (*r* = 0.77), while the average strength for uric acid (*r* = 0.52) and catalase (*r* = −0.44). As can be seen on the given factor plane (Figure 2a), this set of indicators makes it possible to distinguish between a control group and a group of patients with diseases of the oral cavity, however, in this way it is not possible to differentiate between benign and malignant pathologies.

**Figure 3:**
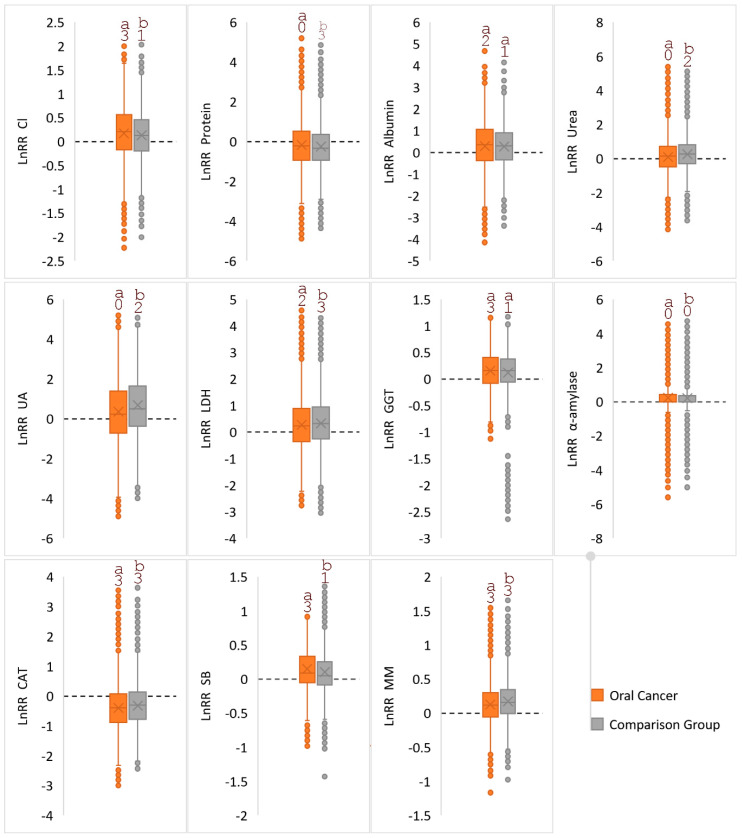
The intensity of the difference (*Ln*RR = *Ln*(OSCC/Control) and *Ln*RR = *Ln*(Comparison/Control)) by 11 biochemical parameters from the control group. Similar letters do not differ much (α = 0.05). The dashed line represents the zero level (*Ln*RR = 0). Significance of differences from 0 was checked using the t-test of one sample and indicated by numbers (“0” is not significant; “1” is *p* < 0.05; “2” is *p* < 0.01; “3” is *p* < 0.001).

If we consider the change in the parameters relative to the control group, then we can note a unidirectional character both for the comparison group and for the oral cancer group (Figure 3).

The presence of outliers is illustrated by the heterogeneity of the observed values, for example, for the LnRR (GGT) in the comparison group, the average value increased statistically significantly, but there are many outliers with decreased values, that is, one cannot speak of a guaranteed increase in the values of this indicator, even having confirmation in the control group. One of the probable reasons may be the combination of all stages of oral cancer in one group; therefore, at the next stage of the study, the main group was further divided into stages (Table 2).

In an attempt to separate the oral cancer group by stages, the PCA-analyzed data contained 66 records, 24 variables each, of which 19 quantitative variables are illustrative and 1 qualitative variable is illustrative, which is quite representative. The first two measurements cover 81.98% of the total inertia of the data set (46.10% of the first axis and 35.88% of the second axis); this means that 81.98% of the total variability is explained by two PCs. This percentage is relatively high and, therefore, the two-dimensional plane well represents the variability of the data. It follows that more than 1/3 of all observations will be well interpreted. As a result of PCA, we obtained the distribution of parameters along the axes of the factor plane (Figure 2c,d; *p* < 0.001). For PC1 (horizontal axis), high correlation coefficients were obtained for Schiff bases (*r* = 0.83) and triene conjugates (*r* = 0.79), average strength correlation coefficients for albumin (*r* = 0.51), urea (*r* = 0.51), potassium (*r* = 0.51), catalase (*r* = 0.47), protein (*r* = 0.47) and LDH (*r* = 0.40). For PC2 (vertical axis), high correlation coefficients correspond to potassium (*r* = 0.72) and albumin (*r* = 0.72); average correlation strengths were obtained for chlorides (*r* = 0.63), protein (*r* = 0.57), uric acid (*r* = 0.53), GGT (*r* = 0.45), sodium (*r* = 0.42), Schiff bases (*r* = −0.41) and triene conjugates (*r* = −0.49). Nevertheless, as can be seen in the above figures, only stages I–III and stage IV can be separated between each other (Figure 2c,d).

**Figure 4:**
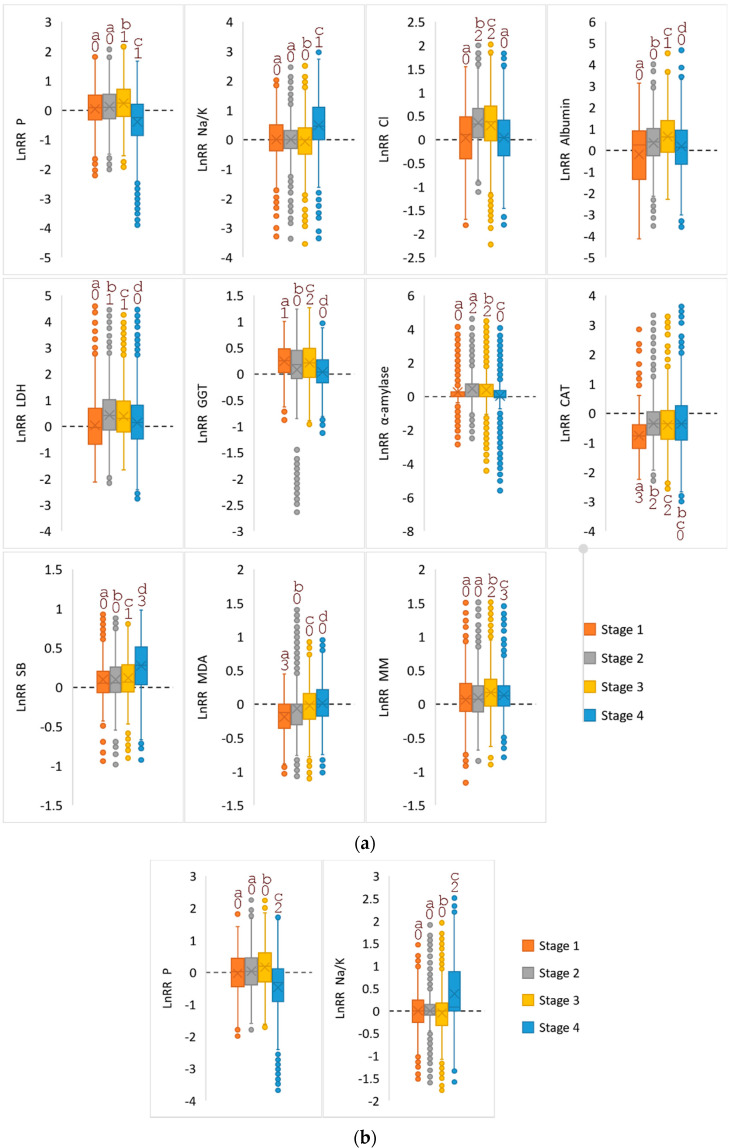
The intensity of the difference *Ln*RR = *Ln*(OSCC/Control) for 11 parameters relative to the control group according to stages (**a**) and *Ln*RR = *Ln*(OSCC/Comparison) for 2 parameters relative to the comparison group (**b**). Similar letters do not differ much (α = 0.05). The dashed line represents the zero level (*Ln*RR = 0). Significance of differences from 0 was checked using the t-test of one sample and indicated by numbers (“0” is not significant; “1” is *p* < 0.05; “2” is *p* < 0.01; “3” is *p* < 0.001).

The calculation of the Kruskal–Wallis N-test values showed that the separately taken stages of oral cancer and the control group reliably differ in a fairly large number of indicators (Table 3), while comparing the same stages with the comparison group shows differences in only two indicators (phosphorus and Na/K-ratio, Table 3). Charts were constructed based on significantly different indicators (Figure 4a,b), which show the change in the considered indicators depending on the stage of the disease.

The calculations showed that none of the considered indicators in their own version could be used to diagnose oral cancer; however, a combination of a number of indicators allows us to differentiate the groups among themselves. So, according to the parameters “albumin + protein + MM + uric acid” (Figure 2), it is possible to distinguish the combined group (OSCC + Comparison Group) from the control group, and to separate the stages among themselves inside the OSCC group, you need to use other parameters of the “Schiff base + triene conjugates + potassium + albumin ”(Figure 4). However, it is not possible to distinguish between stages of oral cancer I–III and non-malignant diseases of the oral cavity using only the parameters considered.

### 3.2. Prognostic Value of Biochemical Saliva Markers in OSCC

**Figure 5:**
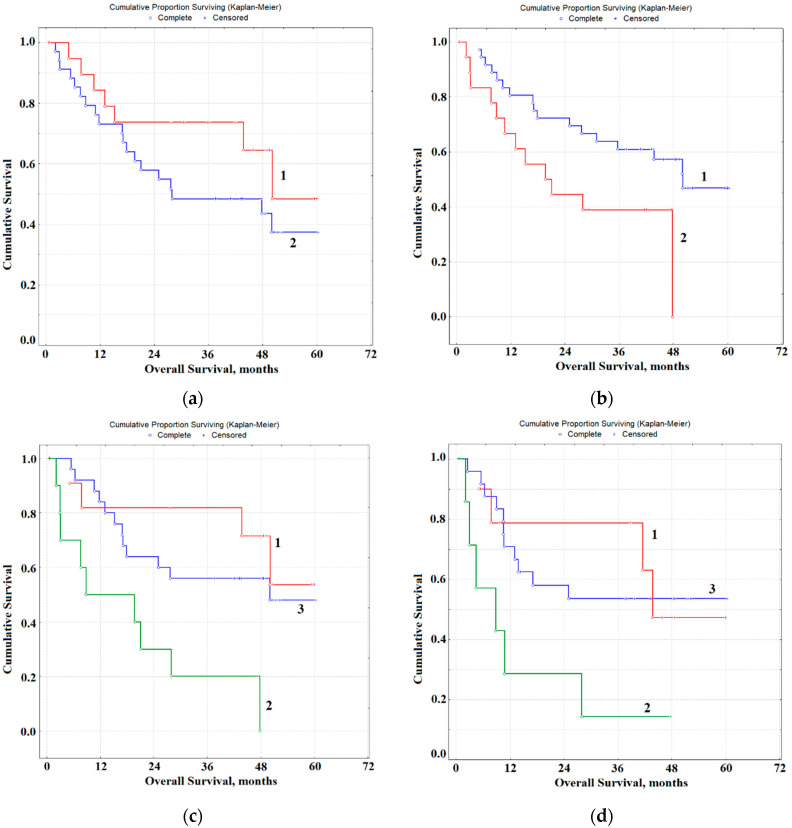
The overall survival of OSCC patients depending on the concentration of MDA and Na/K ratio in saliva: (**a**) MDA > 7.34 nmol/mL (curve 1) and MDA < 7.34 nmol/mL (curve 2); (**b**) Na/K < 1.09 c.u. (curve 1) and Na/K > 1.09 c.u. (curve 2); (**c**) multivariate regression analysis of overall survival: MDA > 7.34 nmol/mL and Na/K < 1.09 c.u. (curve 1); MDA < 7.34 nmol/mL and Na/K > 1.09 c.u. (curve 2); other combinations (curve 3); (**d**) multivariate regression analysis of the disease-free survival: MDA > 7.34 nmol/mL and Na/K < 1.09 c.u. (curve 1); MDA < 7.34 nmol/mL and Na/K > 1.09 c.u. (curve 2); other combinations (curve 3).

For the study group, the median of overall survival (OS) was 30.4 months, and disease-free survival (DFS) was 16.0 months. According to the results of multivariate regression analysis, it was found that the concentration of malondialdehyde (MDA) and the Na/K-ratio in saliva are associated with survival rates for OSCC patients (Figure 5a,b).

The average values for the OSCC group (for MDA—7.34 nmol/mL, for Na/K—1.09 c.u.) were used as threshold values for indicators of OS. The MDA concentration of more than 7.34 nmol/mL (*p* = 0.17155) and the Na/K-ratio of less than 1.09 c.u. are predictively favorable (*p* = 0.06187, Table 4). The combination of both parameters is a more effective prognostic sign (*p* = 0.01876, Table 4). For patients with a favorable prognosis (MDA > 7.34 and Na/K < 1.09), the 1-year overall survival rate decreases sharply when switching from a favorable to an unfavorable prognosis from 81.8 to 50.1%, 3-year from 81.8 to 20.1%, 5-year-old from 53.7 to 0% (Figure 5c). The combination of intermediate values in terms of survival is closer to the group with a favorable prognosis; the differences between the groups are statistically unreliable. For a combination of parameters, indicators of DFS were additionally calculated (Figure 5D). In this case, median of DFS with a favorable prognosis was 40.7 months, with an unfavorable prognosis—6.7 months, in all other cases—21.4 months (HR = 4.20, 95% CI 0.74–23.24, *p* = 0.05775).

## 4. Discussion

It was established that against the background of cancer and benign pathologies of the oral cavity, a change in the metabolic profile of saliva occurs. Largely, the changes relate to protein metabolism: the total protein content decreases, but the content of albumin, urea and uric acid increases (Table 1). The activity of metabolic enzymes (LDH, GGT and α-amylase) increases, while the activity of antioxidant enzymes, in particular catalase, decreases, and the content of toxic metabolites: Schiff bases and MM increases. Among electrolyte components, only an increase in chloride concentration is statistically significant. The observed changes as a whole fit into the picture of metabolic changes taking place against the background of oncological processes in the human body. In oral cancer, because of genetic changes, significant changes occur in critical metabolic pathways, such as glycolysis, tricarboxylic acid cycle, pentose phosphate cycle, polyamine synthesis, urea metabolism, etc. [41,42]. The imbalance of the lipid peroxidation system and antioxidant protection of saliva occurs, the activity of the enzymatic link decreases (catalase), and the role of the non-enzymatic link (albumin, uric acid) increases. Antioxidant saliva compounds are closely associated with oral cancer, and the oxidative radicals of saliva definitely reflect the stage of oral cancer and can be used to detect it in the early stages [43]. It was previously found that the content of carbonyl derivatives significantly increases in the saliva of OSCC patients, due to the direct effect of free radicals on saliva proteins, which is an indicator of oxidative damage to proteins [44]. Because of oxidative damage, protein toxins accumulate in saliva, which is expressed in a significant increase in the MM content against the background of a decrease in the total protein content (Table 1 and Table 2). MDA is the final product of lipid peroxidation; its content is also used to characterize the degree of oxidation and antioxidant imbalance [45]. The level of MDA in saliva showed a statistically significant increase in the groups of precancerous diseases and cancer. MDA itself is also highly toxic and can cause carcinogenesis in the oral cavity [46]. As a convenient oxidation marker for studying the antioxidant potential of saliva, a general antioxidant capacity (AOC) is proposed [47]. The AOC of saliva decreases with precancerous diseases of the oral cavity, such as leukoplakia, lichen planus, erythroplakia, and oral cancer [32]. In our case, it was shown that the content of the main enzyme of the antioxidant protection of saliva, catalase, decreases, but the activity of superoxide dismutase does not significantly change, while for the non-enzymatic link of antioxidant protection, even an increase in concentrations is observed. Apparently, in this case, compensatory mechanisms are included in the work, and therefore, to understand the processes that occur against the background of oral cancer, it is necessary to consider not generalized indicators, but individual links of the saliva antioxidant defense system, in particular, catalase [48].

It is noted that with oral cancer, the activity of metabolic enzymes of saliva significantly increases. Earlier in the literature, this was noted only for LDH, whose activity is increased in the saliva of patients with leukoplakia and OSCC [49,50]. An increase in LDH activity may be due to the intensification of the production of lactate from pyruvate under anaerobic conditions, which is a key feature of cancer cells [51]. The important role of the glutathione system in antioxidant defense is known; GGT-dependent prooxidant reactions are involved in the inhibition of cancer cell proliferation [52,53]. The revealed increase in the activity of α-amylase according to published data can also be a diagnostically important sign; however, the presence of a large number of outliers (Figure 3) indicates the heterogeneity of the distribution of this parameter within the studied groups and its low reliability [54].

Against the background of OSCC, according to some data, the author noted an increase in the level of calcium, inorganic phosphate, magnesium, and sodium and a decrease in the level of potassium [55]. A change in the ionic composition may be associated with oral dehydration of OSCC [33,56]; however, in this case, a change in the salivation rate of saliva and an increase in the concentration of other components, for example, protein, would also be noted. The increase in the content of electrolyte components may be associated with a violation of the structural properties of saliva and changes in protein metabolism against pathological processes in the oral cavity. Based on the theory of the micellar structure of saliva, electrolyte components are part of the diffuse layer and stabilize calcium phosphate micelles, but they can be released when the balance is unbalanced [39]. This process should be considered as secondary, since statistically significant differences between the groups were revealed only for chlorides. Nevertheless, in recent decades, more and more scientific data confirm the role of ion channels in the development of various forms of cancer [57]. Both potassium-selective pores and chloride permeability are considered the most active channels in oncogenesis. High levels of proliferation, active migration, and invasion into non-tumor tissues are specific properties of neoplastic transformation. All these actions require a partial or full participation of the activity of the chloride channel. Another study showed that chloride ions play an important role in the progression of the cell cycle by regulating the expression of p21 via the p53-independent pathway in human gastric cancer cells [58]. So, in the Cl-substituted medium (replacement of chloride ions by nitrate ions), a decrease in the intracellular concentration of chlorides occurred and inhibition of cell growth was observed due to the stop of the cell cycle in the G0/G1 phase. Thus, the likely role of chlorides in the development of oral cancer may be wider, which requires further research in this direction.

When taking into account the stage of the disease, lipid peroxidation products come to the fore, the level of which increases significantly in the advanced stages of the disease (Table 2). It was shown by PCA that only stage IV disease can be isolated using the described parameters. Thus, it was found that the saliva of patients in the control group was significantly different from the saliva of patients with oral pathologies, while patients with non-cancerous pathologies and patients with stages I–III of OSCC can be differentiated only from advanced stages of the disease.

It is interesting to note that the metabolic changes occurring in saliva against the background of oral cancer can be used to predict the outcome of the disease (Table 4). Perhaps the use of MDA for these purposes and the Na/K-ratio of saliva are not described in the literature. The poor prognosis and early recurrence of OSCC is known to correlate with the simultaneous high levels of C-reactive protein (CRP), cancer-embryonic antigen (CEA) and squamous cell carcinoma antigen (SCCA), elevated white blood cell count, absolute neutrophil and platelet count, low expression of Caspase-3, high expression of p53 and bcl-2, and no reduction in Ki-67 expression in tumor tissue. A decrease in the Ki-67 index at the treatment stages, high expression of Caspase-3, low expression of bcl-2 and p53, low levels of CRP, CEA and SCCA, and HPV-positive tumor status are associated with a favorable prognosis of the course of the tumor process [35]. The use of miRNAs as prognostic biomarkers in OSCC has been described [59]. The possibility of using the CRP/albumin ratio to determine the prognosis of OSCC has been shown [24]. The group with a high CRP/albumin ratio (>0.085) had a higher clinical stage of TNM (*p* = 0.002) and larger primary tumors (*p* = 0.029) with statistically significant differences in lymph node metastasis and distant metastasis. In addition, when the CRP/albumin ratio was high, multivariate analysis showed lower survival (*p* = 0.002; HR = 6.078). However, all these indicators are determined in the tumor tissue by the immunohistochemical method or in blood serum, while the use of saliva for prognostic purposes can be more convenient and informative. At the same time, we obtained comparable values of the risk ratio (HR = 7.88, 95% CI 1.10–54.62, *p* = 0.01876), which allows us to use the proposed indicators (MDA and Na/K-ratio) for prognostic purposes in oral cancer.

The indicators selected using multivariate regression analysis statistically significantly change with the progression of OSCC (Table 3). However, they are not each independent prognostic parameters; a high HR is achieved only with a combination of MDA and the Na/K-ratio (Table 4). A detailed analysis of the data of patients revealed that for the group of favorable prognoses, the proportion of patients with early stages of OSCC is slightly higher than for the group with unfavorable prognosis (38.9 vs. 23.8%). The proportion of advanced cases (stage IV) is, on the contrary, lower in the first case (20.5 vs. 61.9%). However, the high prognostic value of MDA in combination with the Na/K-ratio can be unambiguously explained only after additional studies.

Limitations of this study are associated with a small sample size, and therefore the early stages of the disease are worse than advanced. As a comparison group, a group of patients hospitalized for the surgical treatment of neoplasms that needed to be differentiated from malignant was selected, but patients with premalignant lesions (leukoplakia) should be included in the next stage of the study. In addition, understanding the variability of saliva composition between patients, the differentiation between patients with chronic inflammatory diseases of the oral cavity or other types of cancer, as well as the standardization of the collection, processing and storage of saliva remain issues that need clarification.

## 5. Conclusions

In the course of the study, metabolic features of the composition of saliva in OSCC were revealed. It was shown that none of the studied parameters can be used to diagnose OSCC in an independent variant; however, the use of combinations of parameters is more informative. The high prognostic value of the MDA content and the Na/K-ratio in saliva was established before treatment. Thus, the content of MDA ˂ 7.34 nmol/mL and the Na/K-ratio > 1.09 c.u. is a prognostically unfavorable factor (HR = 7.88, 95% CI 1.10–54.62, *p* = 0.01876), which may be useful for optimizing the treatment of patients with OSCC.

## Figures and Tables

**Table 1:** The results of biochemical analysis of the studied groups.

Indicators	Control Group, n = 114 (1)	Comparison Group, n = 50 (2)	Oral Cancer, n = 68 (3)	Kruskal–Wallis Test (*H*, *p*)
**Flow rate, mL/min**	0.87 (0.72; 0.99)	0.81 (0.74; 0.94)	0.78 (0.64; 0.97)	1.079, 0.5836
**Electrolytes**				
**Calcium, mmol/L**	1.26 (1.03; 1.61)	1.38 (1.10; 1.69)	1.39 (1.14; 1.85)	4.599, 0.1003
	-	-	p_1–3_ = 0.0426	
**Phosphorus, mmol/L**	4.36 (3.39; 5.58)	4.70 (3.62; 6.38)	4.29 (3.03; 6.19)	1.350, 0.5091
**Ca/P**	0.290 (0.226; 0.399)	0.267 (0.199; 0.474)	0.316 (0.241; 0.601)	2.762, 0.2514
**Sodium, mmol/L**	7.5 (4.9; 11.2)	7.6 (5.3; 10.9)	9.2 (6.3; 13.9)	4.222, 0.1211
	-	-	p_1–3_ = 0.0403	
**Potassium, mmol/L**	10.5 (8.6; 13.3)	12.0 (8.9; 15.6)	11.0 (8.3; 16.1)	2.291, 0.3181
**Na/K**	0.68 (0.48; 1.08)	0.67 (0.49; 1.04)	0.88 (0.59; 1.40)	5.575, 0.0616
	-	-	p_1–3_ = 0.0220	
**Chlorides, mmol/L**	23.7 (19.4; 29.6)	26.1 (21.7; 33.9)	30.1 (21.9; 39.5)	12.38, 0.0020 *
	-	p_1–2_ = 0.0325	p_1–3_ = 0.0009	
**Protein Metabolism**				
**Protein, g/L**	1.11 (0.78; 1.53)	0.75 (0.49; 1.23)	0.84 (0.47; 1.59)	8.581, 0.0137 *
	-	p_1–2_ = 0.0051	-	
**Albumin, g/L**	0.24 (0.17; 0.36)	0.34 (0.19; 0.48)	0.36 (0.19; 0.67)	11.53, 0.0031 *
	-	p_1–2_ = 0.0108	p_1–3_ = 0.0031	
**Urea, mmol/L**	6.76 (4.35; 8.78)	8.67 (5.73; 13.48)	8.66 (4.62; 11.29)	7.823, 0.0200 *
	-	p_1–2_ = 0.0072	-	
**Uric acid, nmol/mL**	76.8 (25.2; 161.2)	124.5 (67.3; 193.6)	93.0 (43.2; 182.9)	9.346, 0.0093 *
	-	p_1–2_ = 0.0028	-	
**Sialic acids, mmol/L**	0.189 (0.134; 0.311)	0.186 (0.131; 0.250)	0.220 (0.140; 0.336)	1.147, 0.5634
**Enzymes**				
**ALP, U/L**	60.84 (39.11; 84.75)	70.62 (47.81; 99.96)	73.88 (49.98; 109.74)	5.128, 0.0770
	-	-	p_1–3_ = 0.0439	
**LDH, U/L**	1008.0 (607.9; 1702.0)	1471.0 (1121.0; 2098.0)	1441.0 (864.8; 2028.0)	11.30, 0.0035 *
	-	p_1–2_ = 0.0026	p_1–3_ = 0.0153	
**GGT, U/L**	18.6 (15.4; 23.0)	22.4 (19.7; 24.7)	23.2 (17.9; 27.0)	21.45, 0.0000 *
	-	p_1–2_ = 0.0002	p_1–3_ = 0.0001	
**α-amylase, U/L**	178.8 (74.3; 382.9)	316.1 (127.2; 503.7)	315.1 (196.3; 519.5)	8.345, 0.0154 *
	-	-	p_1–3_ = 0.0100	
**Catalase, mcat/L**	4.52 (3.60; 5.90)	3.48 (2.34; 5.13)	3.00 (2.18; 4.56)	27.79, 0.0000 *
	-	p_1–2_ = 0.0005	p_1–3_ = 0.0000	
**SOD, c.u.**	61.8 (36.8; 115.8)	63.2 (28.9; 131.6)	68.4 (26.3; 123.7)	0.0521, 0.9743
**Lipoperoxidation Products and Endogenous Intoxication Rates**				
**Diene Conjugates, c.u.**	3.90 (3.74; 4.02)	3.94 (3.78; 4.11)	3.93 (3.76; 4.16)	3.259, 0.1960
**Triene Conjugates, c.u.**	0.885 (0.816; 1.042)	0.911 (0.810; 1.080)	0.923 (0.837; 1.047)	0.5642, 0.7542
**Schiff Bases, c.u.**	0.534 (0.494; 0.570)	0.545 (0.497; 0.653)	0.558 (0.513; 0.715)	12.95, 0.0015 *
	-	-	p_1–3_ = 0.0004	
**MDA, nmol/mL**	6.92 (6.15; 9.06)	6.84 (5.73; 8.72)	6.58 (5.56; 7.78)	2.716, 0.2572
**MM 280/254 nm**	0.778 (0.694; 0.878)	0.918 (0.818; 1.036)	0.895 (0.795; 0.990)	30.09, 0.0000 *
	-	p_1–2_ = 0.0000	p_1–3_ = 0.0000	

Note. p_1–2_, p_1–3_—statistically significant differences compared with control group (Mann–Whitney U-criterion); *H*—Kruskal–Wallis criterion; *p*—significance level; *—differences are statistically significant, *p* ˂ 0.05.

**Table 2:** Biochemical indicators of saliva in OSCC, depending on the stage I–IV.

Indicator	St I, n = 10	St II, n = 16	St III, n = 20	St IV, n = 22
**Flow rate, mL/min**	0.80 (0.67; 0.95)	0.78 (0.62; 0.98)	0.81 (0.70; 1.01)	0.76 (0.62; 0.93)
**Electrolytes**				
**Calcium, mmol/L**	1.23 (1.00; 1.45)	1.52 (1.31; 2.24)	1.55 (1.20; 1.86)	1.27 (0.79; 1.94)
	-	*p* = 0.0160	*p* = 0.0492	-
**Phosphorus, mmol/L**	4.40 (3.80; 7.13)	4.43 (3.61; 6.82)	5.19 (3.83; 8.64)	3.56 (1.92; 5.50)
	-	-	*p* = 0.0394	*p* = 0.0255
**Ca/P**	0.238 (0.203; 0.276)	0.375 (0.215; 0.550)	0.275 (0.185; 0.568)	0.441 (0.254; 0.861)
	*p* = 0.0002	*p* = 0.0001	*p* = 0.0000	*p* = 0.0191
**Sodium, mmol/L**	14.9 (4.5; 16.1)	8.6 (6.5; 11.9)	7.1 (5.4; 10.9)	10.0 (6.0; 14.0)
**Potassium, mmol/L**	14.4 (10.5; 18.6)	14.1 (8.5; 17.7)	13.3 (9.0; 16.5)	9.2 (4.0; 15.6)
	*p* = 0.0376	-	-	-
**Na/K**	0.810 (0.386; 1.198)	0.668 (0.598; 0.917)	0.733 (0.327; 0.840)	1.282 (0.880; 1.984)
	*p* = 0.0005	*p* = 0.0000	*p* = 0.0000	*p* = 0.0000
**Chlorides, mmol/L**	27.3 (16.2; 38.7)	31.7 (27.3; 40.0)	35.1 (25.8; 45.5)	24.5 (20.7; 32.9)
	-	*p* = 0.0004	*p* = 0.0007	-
**Protein Metabolism**				
**Protein, g/L**	0.68 (0.55; 1.32)	0.87 (0.53; 1.63)	0.94 (0.44; 1.71)	0.84 (0.45; 1.31)
**Albumin, g/L**	0.39 (0.10; 0.57)	0.39 (0.22; 0.53)	0.48 (0.25; 0.91)	0.35 (0.15; 0.57)
	-	*p* = 0.0353	*p* = 0.0022	-
**Urea, mmol/L**	8.71 (5.09; 13.39)	9.21 (4.98; 13.94)	7.90 (5.08; 10.30)	8.02 (3.02; 10.22)
**Uric acid, nmol/mL**	106.85 (41.83; 187.51)	102.16 (56.23; 188.14)	117.31 (35.31; 192.48)	71.96 (40.00; 136.11)
**Sialic acids, mmol/L**	0.244 (0.150; 0.375)	0.317 (0.098; 0.336)	0.269 (0.174; 0.391)	0.165 (0.119; 0.229)
**Enzymes**				
**ALP, U/L**	94.53 (33.68; 123.86)	78.23 (64.10; 139.07)	59.76 (47.81; 98.87)	72.80 (54.33; 110.82)
**LDH, U/L**	1094.8 (586.5; 1546.0)	1903.5 (1226.5; 2316.5)	1756.0 (938.7; 2179.0)	1295.5 (702.9; 1928.0)
	-	*p* = 0.0151	*p* = 0.0248	-
**GGT, U/L**	23.7 (22.9; 28.8)	24.9 (17.3; 27.4)	24.6 (17.1; 28.9)	19.8 (17.9; 23.9)
	*p* = 0.0105	*p* = 0.0256	*p* = 0.0052	-
**α-amylase, U/L**	272.1 (183.2; 545.9)	463.8 (279.2; 756.0)	422.1 (265.7; 1073.0)	261.6 (106.1; 399.2)
	-	*p* = 0.0096	*p* = 0.0164	-
**Catalase, mcat/L**	2.35 (1.44; 2.69)	3.00 (2.50; 4.73)	3.45 (1.94; 4.61)	3.03 (1.85; 6.39)
	*p* = 0.0001	*p* = 0.0040	*p* = 0.0045	*p* = 0.0178
**SOD, c.u.**	55.3 (23.7; 182.9)	71.1 (28.9; 150.0)	65.8 (25.0; 128.9)	67.1 (30.3; 88.2)
**Lipoperoxidation Products and Endogenous Intoxication Rates**				
**Diene Conjugates, c.u.**	3.88 (3.53; 4.16)	3.87 (3.64; 4.11)	4.09 (3.85; 4.26)	3.86 (3.75; 4.04)
	-	-	*p* = 0.0091	-
**Triene Conjugates, c.u.**	0.919 (0.760; 1.081)	0.886 (0.837; 0.960)	0.923 (0.825; 1.015)	1.011 (0.882; 1.181)
	-	-	-	*p* = 0.0093
**Schiff Bases, c.u.**	0.556 (0.514; 0.621)	0.546 (0.517; 0.680)	0.554 (0.512; 0.691)	0.699 (0.536; 0.907)
	-	-	-	*p* = 0.0000
**MDA, nmol/mL**	5.98 (5.21; 6.32)	5.38 (5.13; 7.61)	7.09 (6.15; 8.03)	7.18 (6.41; 8.97)
	*p* = 0.0175	*p* = 0.0391	-	-
**MM 280/254 nm**	0.930 (0.576; 1.045)	0.839 (0.730; 0.957)	0.937 (0.784; 1.039)	0.901 (0.818; 0.924)
	-	-	*p* = 0.0018	*p* = 0.0022

Note. *p—*Statistically significant differences compared with control group (Mann–Whitney U-criterion).

**Table 3:** The values of the Kruskal–Wallis criterion when comparing patients with OSCC by stages with a control group and a comparison group.

Indicators	Kruskal–Wallis Test (*H*, *p*)	
	Control Group, n = 114	Comparison Group, n = 50
**Electrolytes**		
**Calcium, mmol/L**	8.728, 0.0683	4.776, 0.3111
**Phosphorus, mmol/L**	10.72, 0.0299 *	10.41, 0.0341 *
**Ca/P**	7.739, 0.1016	6.252, 0.1811
**Sodium, mmol/L**	5.818, 0.2140	2.588, 0.6289
**Potassium, mmol/L**	8.750, 0.0677	4.840, 0.3041
**Na/K**	13.19, 0.0104 *	12.58, 0.0135 *
**Chlorides, mmol/L**	20.41, 0.0004 *	8.900, 0.0637
**Protein Metabolism**		
**Protein, g/L**	3.459, 0.4842	1.726, 0.7859
**Albumin, g/L**	12.01, 0.0173 *	3.487, 0.4799
**Urea, mmol/L**	4.783, 0.3016	1.164, 0.8063
**Uric acid, nmol/mL**	2.906, 0.5738	1.972, 0.7410
**Sialic acids, mmol/L**	6.715, 0.1518	7.317, 0.1201
**Enzymes**		
**ALP, U/L**	5.660, 0.2260	1.582, 0.8121
**LDH, U/L**	10.19, 0.0374 *	4.283, 0.3690
**GGT, U/L**	16.42, 0.0025 *	6.669, 0.1545
**α-amylase, U/L**	12.41, 0.0145 *	6.313, 0.1770
**Catalase, mcat/L**	28.05, 0.0000 *	4.612, 0.3294
**SOD, c.u.**	0.2268, 0.9940	0.2448, 0.9931
**Lipoperoxidation Products and Endogenous Intoxication Rates**		
**Diene Conjugates, c.u.**	7.406, 0.1159	6.668, 0.1545
**Triene Conjugates, c.u.**	8.209, 0.0842	7.210, 0.1252
**Schiff Bases, c.u.**	20.59, 0.0004 *	6.190, 0.1854
**MDA, nmol/mL**	9.623, 0.0473 *	7.446, 0.1141
**MM 280/254 nm**	17.65, 0.0014 *	3.365, 0.4987

Note. *H*—Kruskal–Wallis criterion; *p*—significance level; *—differences are statistically significant, *p˂0.05*.

**Table 4:** Analysis of biochemical saliva parameters affecting overall survival of OSCC patients.

Indicators	Category	HR (95% CI)	*p*-Value	OS, Months
**MDA, nmol/mL**	˂7.34, n = 42	1	0.17155	25.4
	>7.34, n = 26	0.45 (0.15–1.37)		36.8
**Na/K, c.u.**	˂1.09, n = 39	1	0.06187	44.0
	>1.09, n = 29	1.49 (0.51–4.36)		15.5
**MDA + Na/K**	Favorable prognosis, n = 16	1	0.01876	49.3
	Other combinations, n = 35	1.40 (0.33–5.86)		38.1
	Unfavorable prognosis, n = 17	7.88 (1.10–54.62) *		9.0

Note. OS—overall survival; *—differences are statistically significant, *p* < 0.05.
